# Functional ambulation without lower-leg muscles or nerves — a case report with video

**DOI:** 10.1080/17453674.2019.1642432

**Published:** 2019-07-18

**Authors:** John Parenti

**Affiliations:** Department of Orthopaedic Surgery, Geisinger Medical Center, Danville, PA, USA

Our orthopedic service was called for an urgent consultation concerning a 56-year-old man who had been admitted to Intensive Care some 36 hours earlier because of syncope, cyanosis, profound orthostatic hypotension, severe hemoconcentration, and acute bilateral leg pain. His medical history was unremarkable, and the tentative diagnosis given was polycythemia of unknown etiology leading to venous thromboembolism, with a distributive shock pattern consistent with sepsis. In the wake of aggressive IV hydration while intubated, swelling and tenseness had developed in his lower extremities secondary to massive thrombosis in all limbs.

The patient’s pressures were greater than 60 mmHg in all 4 compartments of both lower extremities, and they were elevated also in his forearms. Bilateral lower-extremity fasciotomies were performed immediately, and after the fascia were incised the muscles were poorly contractile and there was evidence of developing necrosis. 2 subsequent interventions in the days that followed entailed staged debridements to remove some necrotic musculature from both anterior and lateral lower-leg compartments.

Based on the patient’s symptoms and clinical findings, the ICU staff eventually considered an alternative diagnosis of systemic capillary leak syndrome (SCLS). SCLS is a rare episodic disease of unknown etiology characterized by self-reversing episodes during which the endothelial cells that line the capillaries, usually in the extremities, separate for up to 3 days, causing a leakage of plasma mainly into the muscle compartments of arms and legs (Druey and Parikh 2017). The extravasation can be sufficiently massive to cause circulatory shock and compartment syndrome, especially when undiagnosed SCLS patients are treated as septic and are resuscitated aggressively, as happened here.

In the span of 1 week, the patient recovered hemodynamic stability and his venous clots dissolved, CPK levels decreased, liver and kidney functions normalized, and peripheral edema resolved, whereupon he was extubated. These developments supported the diagnosis of SCLS. However, given the extent of rhabdomyolysis, our surgical team was uniformly pessimistic on the chances of salvaging the patient’s lower limbs. He was deemed too vulnerable to infection, accidental loss of blood circulation to the feet, and other complications and negative outcomes (e.g., reflex sympathetic dystrophy and paresthesia) of limb salvage in the wake of acute compartment syndrome (von Keudell et al. 2015). Even in a best-case scenario, it was hard to imagine that the patient would be left with functional extremities.

Bilateral above-the-knee amputations appealed to our team for their ability to enhance patient survival, reduce pain and disability, and shorten hospitalization. As per the conventional wisdom, limbs that would likely end up flail, painful, insensate, and nonfunctional would be inferior to an amputation and prosthetic fitting—especially true in the lower extremities, given that modern prosthetics have proven to be effective in the restoration of almost normal function (Russell et al. 1991).

The patient was adamantly against amputation, however, arguing that his overall stabilization and good baseline health would see him through the necessary serial debridements. Since there were pulses in the patient’s feet, his general clinical picture was rapidly improving, and he was relatively young and free of comorbidities, there was no need for an urgent amputation.

Nonaggressive debridements took 9 more surgeries over a 5-week period. At the end, no muscles or nerves survived below either knee, and both feet were immobile and insensate. The patient’s sequelae were pain and swelling in all extremities and major flexion contractures of the hands, because he had also suffered partial muscular and neurological damage in the forearms. He was transferred to an inpatient facility for several weeks of physical and occupational therapy, and additional weeks of outpatient hand therapy and physical rehabilitation followed his discharge to home.

These events took place more than 13 years ago, during the winter of 2005–2006, and the patient is alive and well, with his SCLS under control since 2010. Within 6 months of discharge from the rehabilitation facility in February 2006, he was ambulatory and back to work as a full-time university professor. This entailed the return to a grueling weekly commute between 2 major East Coast cities using public transportation (buses, subways, and trains), while taking care of personal needs entirely on his own. By now he is 69 years old and continues to work and commute.

He walks on paved surfaces with a surprisingly normal gait and takes very natural, long strides that belie his disability (Figure 1). He can ambulate up or down ramps and stairs with little difficulty, though he is justifiably unsteady on soft, uneven, or slippery terrain (e.g., sand or snow). While he cannot run, he does not mind because he had always led a sedentary life and did not play sports. The initial swelling of his feet gradually dissipated with the use of compression stockings, and his limb pain subsided such that he quickly weaned himself from pain medication.

For greater stability while ambulating outdoors, he uses a cane and a discreet, carbon-fiber ankle–foot orthosis for added ankle stability. While in the comfort of his 2 apartments, however, he manages so well that he neither uses the cane nor puts on his braces. Thus, he can get up from bed in the nighttime and visit the bathroom simply by propelling himself forward while maintaining balance.

The patient has taken excellent care of his feet: they are free of deformities, scars, or skin changes. He has never needed to be treated for an infection in either foot. The physical condition of his legs and feet after more than 13 years of continuous, post-traumatic daily use is admirable.

He can be watched ambulating with orthoses at 13 years post-trauma on the internet (https://www.youtube.com/watch?v=We_XXrQUmkw&feature=youtu.be), and without braces or cane (https://www.youtube.com/watch?v=ojB1sk7ARhw&feature =youtu.be).

## Discussion

This patient provides an uplifting example of the power of personal determination, good habits (e.g., he is a non-diabetic, non-smoker), and physical and mental adaptation. We think of him as having been amputated, after all, but as having provided his own prostheses. His ability to balance himself is akin to someone who has learned to walk on stilts. His knees, quadriceps, and hips are evidently doing some of the work that his calves and ankles used to perform, yet without causing any damaging wear and tear.

The patient’s incredible adjustment to otherwise devastating loss is illustrated best by the fact that he has taken to driving his own car once again, having logged post-trauma over 150,000 miles of city and highway driving without incident. Evidently, his knees, quads, and eyes provide him the necessary information to distinguish between accelerator and brake pedals, and to regulate vehicle speed at short notice.

To be sure, we have encountered sedentary patients who are satisfied with a poorly functional salvaged limb they can call their own (Attinger and Brown 2012). But the patient reported here rightly perceives his salvaged limbs to be very functional, and, as they have proven to be biomechanically sound and durable despite the passage of time, he has every reason to be happy with them. With the benefit of hindsight, amputation was not the better choice.

Therefore, his case instigated our department to question the conventional wisdom concerning the lack of function in flail and insensate lower legs, as well as in rigid and insensate feet, to the extent that our service has since embraced the cause of limb salvage. We became much more cautious before making amputation recommendations, and we established a now busy wound-care center dedicated to the cause of limb preservation.

This case is a vivid reminder that the decision to attempt limb salvage or to favor amputation is—and should always be—a patient-centered decision (Fiorito et al. 2012). Outcomes assessed from a patient’s perspective have the potential to be distinctly different from those reached by the treating surgeon (Momoh and Chung 2013). Therefore, we must resist the impulse to advocate solutions that appeal because they are relatively safe and expedient. Surgeons must make earnest efforts to understand patients’ medical history, lifestyle, and recovery potential before making assumptions and recommendations—especially as regards irreversible interventions.

**Figure 1.**
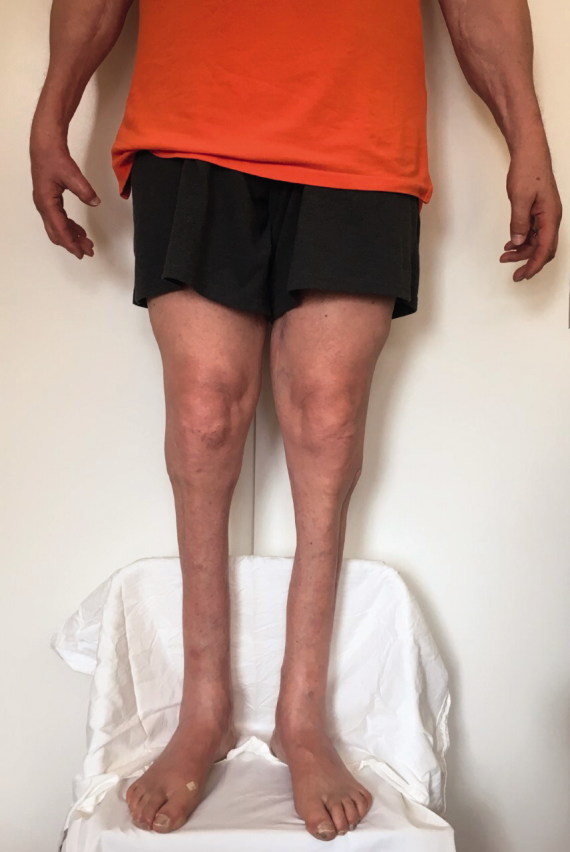
Patient standing at 13 years post-trauma.
